# A comparison of contributions of individual muscle and combination muscles to interaction force prediction using KPCA-DRSN model

**DOI:** 10.3389/fbioe.2022.970859

**Published:** 2022-09-07

**Authors:** Wei Lu, Lifu Gao, Huibin Cao, Zebin Li, Daqing Wang

**Affiliations:** ^1^ Institute of Intelligent Machines, Hefei Institutes of Physical Science, Chinese Academy of Sciences, Hefei, China; ^2^ Science Island Branch, Graduate School of USTC, Hefei, China; ^3^ School of Electrical and Photoelectric Engineering, West Anhui University, Lu’an, China

**Keywords:** surface electromyography, kernel principal component analysis, deep residual shrinkage network, mean impact value, interaction force prediction

## Abstract

Rapid and accurate prediction of interaction force is an effective way to enhance the compliant control performance. However, whether individual muscles or a combination of muscles is more suitable for interaction force prediction under different contraction tasks is of great importance in the compliant control of the wearable assisted robot. In this article, a novel algorithm that is based on sEMG and KPCA-DRSN is proposed to explore the relationship between interaction force prediction and sEMG signals. Furthermore, the contribution of each muscle to the interaction force is assessed based on the predicted results. First of all, the experimental platform for obtaining the sEMG is described. Then, the raw sEMG signal of different muscles is collected from the upper arm during different contractions. Meanwhile, the output force is collected by the force sensor. The Kernel Principal Component Analysis (KPCA) method is adopted to remove the invalid components of the raw sEMG signal. After that, the processed sequence is fed into the Deep Residual Shrinkage Network (DRSN) to predict the interaction force. Finally, based on the prediction results, the contribution of each sEMG signal from different muscles to the interaction force is evaluated by the mean impact value (MIV) indicator. The experimental results demonstrate that our methods can automatically extract the valid features of sEMG signal and provided fast and efficient prediction. In addition, the single muscle with the largest MIV index could predict the interaction force faster and more accurately than the muscle combination in different contraction tasks. The finding of our research provides a solid evidence base for the compliant control of the wearable robot.

## 1 Introduction

In recent years, with the rapid development of robot technology, wearable exoskeleton robots have been favored in many fields, including medical treatment, military, disaster relief, sports, etc (Thanigaivel et al., 2019). Especially in the biomedical field, wearable exoskeleton robot plays an important role in helping the elderly and the disabled. Among them, the control scheme of a robot plays a crucial role in restoring human motor function. However, due to the high flexibility of the human arm, it is difficult to establish its biomechanical model accurately, especially involving interactive torque and force tasks (Guo et al., 2020). Therefore, timely and accurate prediction of the human-computer interaction force has become an important approach to improving compliance control performance (Lu et al., 2021).

sEMG signal is a kind of non-invasive bioelectrical signal which is easy to collect. It is a kind of nonlinear and non-stationary one-dimensional time-series signal generated by the biological current generated when the human surface muscles contract. The amplitude of sEMG is positively correlated with the activation degree of the measured muscles. sEMG signal has attracted attention in the biomedical field due to its advantages of high safety, low cost, and easy operation characteristics. It is worth noting that sEMG signal is generated 30–150 ms ahead of human movement, so it determines that sEMG signal is very suitable for the task of interaction force prediction. Therefore, sEMG signals are more suitable than other signals for interaction force prediction task. Many researchers have also done a lot of studies based on sEMG signals. For example, Zhu and Wang (2020) established a detection method that combined harvester driver fatigue based on ECG and sEMG signals to explore the occurrence and variation of driver fatigue. Wen et al. (2020) realized high-precision grasping force control for deformable objects based on sEMG signal. Ma et al. (2020) collected multi-channel sEMG signals of the forearm and constructed a prediction model with a gene expression programming algorithm (GEP) and BP neural network to predict hand grasping force. Su et al. (2020) realized ankle torque prediction based on surface EMG and angular velocity signal. Nazarpour and Abbasi (2021) used EMG signal and deep regression neural network to estimate the kinematics of knee and ankle joints during squatting training of different intensities.

Currently, researchers have done a lot of studies on force estimation, which include muscle force and interaction force. These methods are mainly divided into the dynamics-based model and muscle information-based model (Chen et al., 2020). Romero and Alonso (2016) improved the force estimation model under different motion forms based on the mechanical expression of the Hill model. Fadzli et al. (2019) used the musculoskeletal model of the right arm of the human upper limb and the developed auxiliary device to estimate the muscle force of an individual. Buchanan et al. (2005) used a forward-reverse dynamics model to estimate muscle force. Chen et al. proposed a finger force prediction method based on sEMG signal and combining the CNN network with the RNN network. The result can effectively realize multi-DOF control of a single finger. Martinez et al. (2020) constructed a grasping force estimation model based on transient EMG by using high-density sEMG. A linear regression function was established to predict grip strength, and fast online sEMG control was realized. In (Huang et al., 2017), the author collects surface sEMG signals based on high-density electrode grids and uses a non-negative matrix decomposition algorithm to process the original signals. Although this method greatly improves the quality of the prediction force and reduces the number of electrodes, it limits the estimation of isometric contraction forces. Na et al. (2017a) proposed a new method to estimate joint forces using biomechanical muscle models and surface sEMG peaks. Youn and Kim (2011) used an artificial neural network model to estimate the relationship between MMG signals under isometric muscle contraction and elbow flexion force. Hua et al. (2021) constructed a set of upper arm pressure estimation frameworks based on armband sEMG signal.

Studies have found that there is another controversial issue for interaction force prediction: some researchers use the form of combined muscles to achieve force prediction. For example, Zhang et al. (2019) selected EMG signals of the biceps brachialis and brachialis of the upper extremity as the research object to estimate the magnitude of joint force. Oboe et al. (2017) constructed an end weight assessment system by collecting EMG signals from eight channels of the upper arm with an armband device. Some researchers chose a single muscle to achieve force estimation. For example, Beck et al. (2004) took a single muscle of biceps brachii as the object to explore the relationship between MMG signal and average power frequency and torque during isometric and isometric contraction activities. Na et al. (2017b) explored the terminal contraction force under fatigue by collecting EMG signals from the dorsal interosseous muscle of the hand. As is well known, muscle contractions are usually performed by multiple skeletal muscles working together. If only one muscle is taken as the research object, it will lead to insufficient information acquisition of movement. If combined muscles are selected for research, it would lead to information redundancy and increased complexity of the algorithm. Therefore, this paper will investigate whether a single muscle or combination of muscles is more suitable for the prediction task. The results can provide effective help for future control algorithm research.

Deep learning algorithms are widely used in the biomedical field, especially in force estimation and prediction. Luo et al. (2019) proposed an RBFNN neural network approach to explore the potential model between EMG signals and human arm forces. Xu et al. (2020) further improved based on Luo and integrated the Hill model with RBFNN neural network to perform accurate estimation of torque estimation and motion intention recognition. Lu et al. (2021) directly collected EMG signals of biceps brachii and input them into the Informer model to predict the contraction force at the elbow end. Li et al. (2021) further improved the contraction form based on the thinking of upper limb contraction force and explored the estimation method of lower limb extension force with the IGWO-SVR algorithm.

PCA is a linear dimension reduction method and KPCA is a nonlinear dimension reduction method when dealing with high dimensional time series signals. The purpose is to reduce the dimension of the signal while minimizing the loss of information. At present, they have been successfully applied in many fields such as machinery, environment, and medicine. The summary is as follows: Peng et al. (2020) combined the KPCA method with the Gaussian Process Regression (GPR) method for the reliability of high-dimensional structures. Alvarez et al. realized climate assessment of different regions of the world based on remote sensing information and nonlinear PCA (CE Fernández and Alfonso-Morales, 2022). It is worth noting that in the applied research in the biomedical field, Wakeling and Rozitis (2004) further explored the relationship between sEMG frequency and motor unit type based on the PCA method. Zou et al. (2020) completed EEG entropy feature extraction based on the fusion method of T-test and KPCA, and realized the identification of drivers’ fatigue driving state. Zhao et al. (2020a) simplified the feature matrix by combining PCA and ICA methods to achieve effective feature extraction and classification tasks of ECG signals. DRSN network adds a soft threshold function based on the ResNet, which can effectively remove the influence of noise-related features on the source signal. Jiang and Liu (2022) proposed a deep learning network framework combining DRSN network and generative adversarial network, which effectively realized the task of deblurring moving images and solved the problems of poor noise immunity and low generalization in the network. Ma et al. (2022) proposed a human gesture recognition method based on EMG signal and deep residual contraction network. Experimental results show that the DRSN-based method is superior to the traditional neural network in recognition accuracy.

In this paper, a novel interaction force prediction method based on the fusion of KPCA and DRSN deep learning framework is proposed to map the relationship between sEMG and interaction force prediction. By combining a deep residual network, soft threshold function, and attention mechanism, redundant information and noise in input signals can be effectively eliminated. Finally, the MIV method was used to evaluate the contribution of muscles corresponding to the input sEMG signals of each channel to force prediction results, and the results were used to explore whether a single muscle or a combination of muscles is more suitable for force prediction tasks. The main contributions of this paper are as follows: 1) The mapping model of EMG signal and interaction force is constructed. 2) The DRSN model is constructed to realize adaptive denoising removal and feature extraction without reference to the manual prior knowledge of the biomechanical model. This method provides a new method for human-computer interaction force prediction. 3) Based on The MIV index, the contribution evaluation model of muscle mass was proposed to explore the muscle distribution affecting the interaction force under different contraction tasks.

## 2 Preliminary

### 2.1 ResNet

**FIGURE 1:**
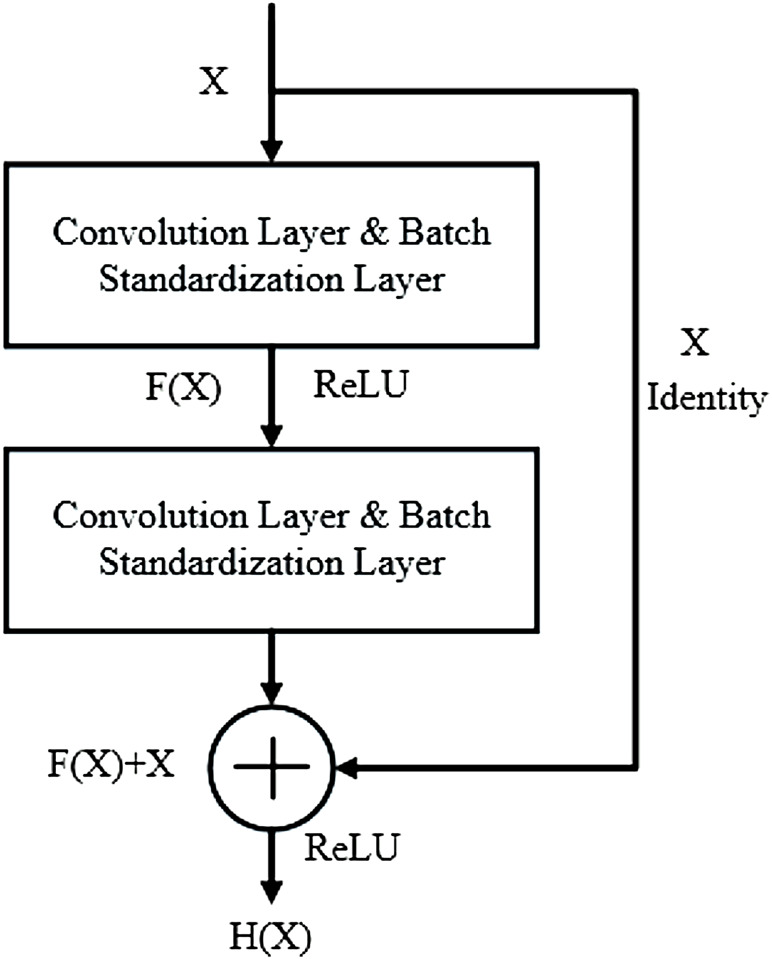
Residual block structure diagram.

In 2016, He et al. proposed an improved feature extraction network based on a convolution neural network, the Deep Residual Network, to solve the problem that the gradient is prone to disappear with the stacking of layers (He et al., 2016; Wang et al., 2022). By introducing several residual structures with direct connection characteristics, the original input information is directly transmitted to the back layer, which solves the problem of gradient disappearance during CNN model training. The algorithm has been successfully applied to image recognition tasks and achieves high accuracy of classification effect. The principle of residual structure is shown in Figure 1.

Suppose the desired optimal solution is H(X)=X , and the residual mapping F(X)= H(X)− X is the residual of H(X) and X . When F(X) is infinitely close to 0, the network is in the optimal state, and the network will still keep in the optimal state if the network depth continues to increase (Yones et al., 2021). When the residual block is input, the calculated output can be:

$$X_{n+1}=f\left(X_{n}+F\left(X_{n},W_{n}\right)\right)$$ (1)

where, F(⋅) is residual mapping, ${\text{W}}_{\text{n}}$ is weight parameter, and f(⋅) is the activation function.

It can be seen that the residual structure has two advantages: 1) The features of the shallow layer can be reused in the deep layer during network forward propagation. 2) In network back propagation, the gradient in the deep layer can be directly transmitted to the shallow layer. Therefore, when there is a large reconstruction error between the input and output of the network, the residual block with a shortcut can directly feedback the error information to the front network layer through the fast connection. This structural design not only improves the model training speed but also effectively alleviates the problem of network degradation.

## 3 Materials and methods

**FIGURE 2:**
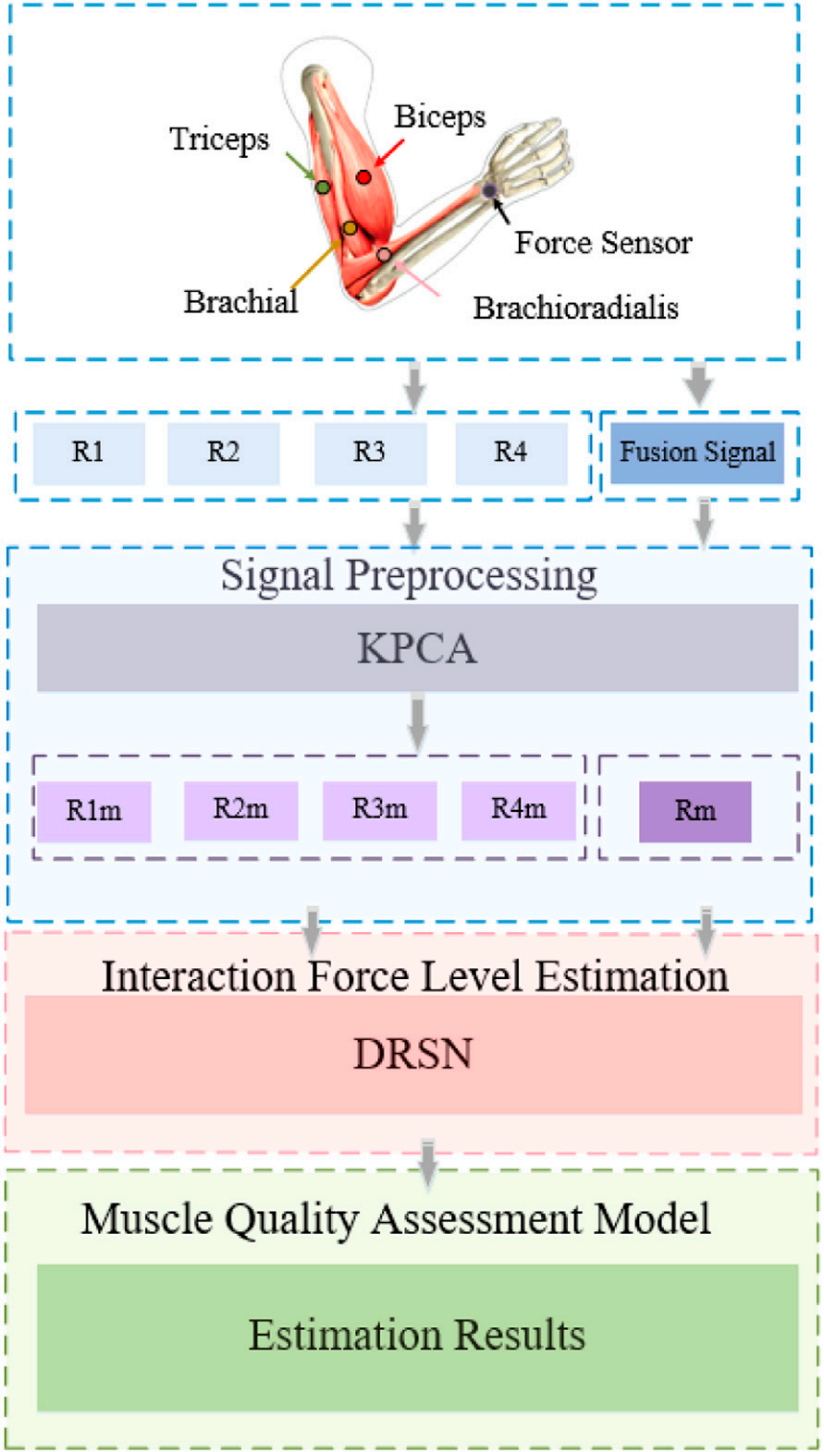
The outline of our approach.

Figure 2 shows the overall research framework of the algorithm proposed in this paper. The purpose of this scheme is to illustrate the mapping between the four muscles of the upper arm (biceps, triceps, brachialis, brachioradialis) and interaction forces. The contribution of each muscle to the prediction of interaction force was evaluated by the MIV index. First, the KPCA algorithm was used to decouple the original signal, and then put them into the DRSN network as tags to complete the interaction force prediction. Based on the analysis of the predicted results, the muscle quality evaluation model was constructed by the MIV index.

### 3.1 KPCA for signal dimension reduction

Since the original surface EMG signals collected have high dimensional characteristics, it is easy to fall into dimensional disaster when extracting effective signal features. In addition, the increasing dimension of data makes the sparsity of data higher. Therefore, it is more difficult to extract effective information from signals. Therefore, it is necessary to combine the high-dimensional variables with correlation into linearly independent low-dimensional variables. Minimize the loss of useful information while compressing information. Principal Component Analysis (PCA), is one of the widest data dimension reduction algorithms. By retaining some important features of high-dimension data and removing noise and unimportant features to achieve the purpose of improving the speed of data processing. The core idea of PCA is to reduce a set of N-dimension vectors to K-dimension, and the goal is to select K unit orthogonal bases so that after the original data is transformed to this set of bases, the covariance between pairs of variables is 0, and the variance of variables is as large as possible. The main steps are as follows:

(1) The original data is composed of matrix X with n rows and m columns according to columns;
(2) Zero-mean processing is performed on each row of X ;
(3) Calculate the covariance matrix of X : $C=\frac{1}{m}XX^{T}$ ;
(4) Calculate the eigenvalues and eigenvectors of the covariance matrix;
(5) The eigenvectors are arranged into a matrix according to the size of eigenvalues in rows from large to small, and the first k rows are selected to form a matrix P .
(6) Y=PX is the data after dimensionality reduction.

Since the eigenvector corresponding to the minimum eigenvalue is often related to noise, the rejection of this part can achieve the effect of denoising. However, PCA has a good effect on linear data processing, but it is hard to process nonlinear data. Therefore, the algorithm has strong limitations. In the next part of this paper, an improved dimension reduction method based on the PCA algorithm is proposed.

The collected sEMG signals are from the sum of all active motion units. As the interaction force increases, the number of active motion units would also increase (Torresguzman et al., 2021). Therefore, it is very difficult to understand and interpret the collected EMG signal accurately because the signal is a nonlinear sequence coupled with the deterministic part and the stochastic part. Therefore, effective analysis of sEMG signals can enable us to accurately understand the mechanism of interaction forces.

Kernel Principal Component Analysis (KPCA) is the nonlinear extension of PCA which is a kind of nonlinear feature extraction method. It can fully mine the nonlinear information contained in data sets. The basic principle is to map the input signal to a high dimensional linear feature space F through a pre-selected nonlinear mapping function ∅ , and then calculate the principal component in the space F using the PCA method. The key point of the algorithm is the selection of the nonlinear mapping function ∅ . The Gaussian kernel is a typical representative of radial basis function, is selected in this paper which is superior to other kernel functions in terms of classification effect (Zhang et al., 2020). The calculation process of the KPCA algorithm can be summarized as follows:

Assuming that the high-dimensional $\left(x_{i}\right)$ input has been averaged and normalized, the covariance matrix can be expressed by:

$$C^{F}=\frac{1}{m}\overset{m}{\underset{i=1}{\sum }}\emptyset \left(x_{i}\right)\emptyset {\left(x_{i}\right)}^{T}$$ (2)

Calculate the eigenvalues and eigenvectors of the matrix ${\text{C}}^{\text{F}}$ , the eigenvectors are denoted as ${\text{v}}_{\text{i}}$ , and the eigenvalues are denoted as ${\text{λ}}_{\text{i}}$ :

$$\lambda v_{i}=C^{F}v_{i};i=1,2,\ldots ,m$$ (3)

Multiply the inner product $\left(x_{i}\right),i=1,2,\ldots ,n$ , on both ends of formula (2), it can be obtained:

$$\lambda_{i}\left(\emptyset \left(x_{i}\right)U\right)=\emptyset \left(x_{i}\right)C^{F}U$$ (4)

Where, $U=\overset{m}{\underset{i=1}{\sum }}\alpha_{i}\emptyset \left(x_{k}\right)$ plug into formula (2) and can be obtained:

$$\lambda \overset{m}{\underset{i=1}{\sum }}\alpha_{i}\left[\emptyset \left(x_{k}\right)\emptyset \left(x_{i}\right)\right]=\frac{1}{m}\overset{m}{\underset{i=1}{\sum }}\alpha_{i}\overset{m}{\underset{j=1}{\sum }}\left[\emptyset \left(x_{k}\right)\emptyset \left(x_{i}\right)\right]\left[\emptyset \left(x_{j}\right)\emptyset \left(x_{i}\right)\right]$$ (5)

Define the kernel matrix as $K_{ij}=\left[\emptyset \left(x_{j}\right)\emptyset \left(x_{i}\right)\right]$ , and Eq. 3 can be simplified as:

nλα=Kα (6)

Where, $\alpha ={\left(\alpha_{1},\alpha_{2},\ldots \alpha_{n}\right)}^{T}$

Let $\bar{\lambda }=n\lambda$ *,* we can get:

$$\bar{\lambda }\alpha =K\alpha$$ (7)

Let $\lambda_{1}\geq \lambda_{2}\geq \ldots \lambda_{m}$ be the non-zero eigenvalue of K , and $\left(\alpha_{1},\alpha_{2},\ldots \alpha_{n}\right)$ be the eigenvector, then the projection of the sample $\left(x_{i}\right)$ in the direction of ${\text{u}}_{\text{k}}$ in higher-dimensional space F is the Kth nonlinear principal component of the sample:

$$h_{k}=\left(U^{k}\cdot \emptyset \left(x\right)\right)=\overset{n}{\underset{i=1}{\sum }}\alpha_{i}^{k}K\left(x_{i},x\right)$$ (8)

The contribution rate of nonlinear principal component ${\text{h}}_{\text{k}}$ to the whole can be obtained by formula (8):

$$\text{Cont}={\text{λ}}_{i}/\overset{N}{\underset{i=1}{\sum }}\lambda_{i}$$ (9)

The larger the Cont value is, the larger the contribution rate of this component is. Major P(P≤N) principal components are selected through the threshold to construct the feature vector after dimension reduction:

$$\text{ξ}=\overset{P}{\underset{i=1}{\sum }}\lambda_{i}/\overset{N}{\underset{i=1}{\sum }}\lambda_{i}$$ (10)

### 3.2 The architecture of DRSN for feature extraction and force prediction

**FIGURE 3:**
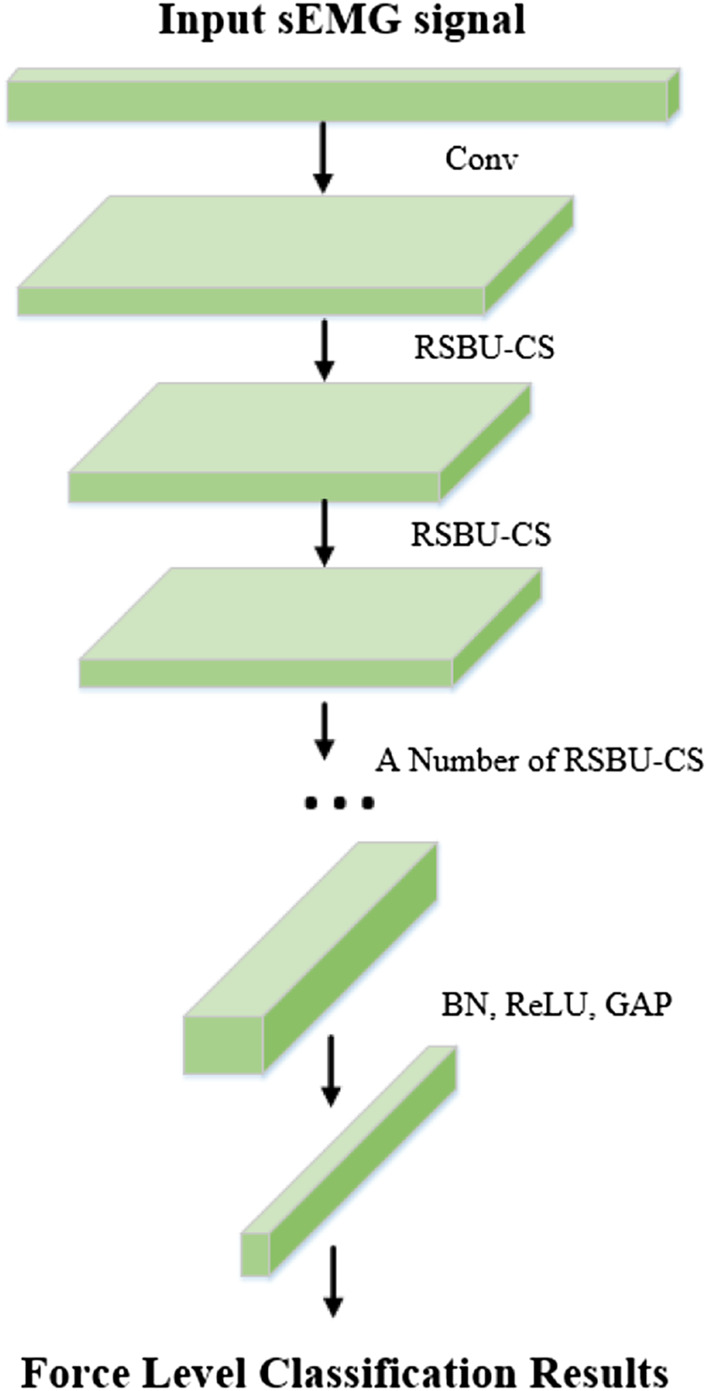
Deep residual shrinkage network structure diagram.

Deep Residual Shrinkage Network (DRSN) was proposed by Zhao et al. (2020b) in 2020 based on ResNet Network, which is a learning method for strong noise or redundant data. The establishment of DRSN is based on three parts: a deep residual network, a soft threshold function, and an attention mechanism. The flow chart of the algorithm is shown in Figure 3.

According to the above analysis, sEMG signal still contains noises of different frequencies and other redundant information although the input signal has been dimensionally reduced. This will still have a great impact on the accuracy of interaction force prediction. In the study of sEMG signal characteristics (Feng et al., 2011; Liu et al., 2013), the feature extraction of sEMG signals is mainly based on manual methods. In this paper, the dimensional-reduction signals are directly fed into the DRSN network, and the features irrelevant to the classification task are zeroed by soft thresholding through the attention mechanism (Diao et al., 2021). The detailed process of the DRSN algorithm is as follows:

#### 3.2.1 ResNet

The input signals are directly mapped to output features, which can be expressed as:

H(x)=F(x)+x (11)

Where F(x) represents the residual if  F(x)=0 , the output and input are identical mappings.

The residual element is calculated by the following formula:

$$y=F\left(x,\left\{W_{i}\right\}\right)+W_{s}x$$ (12)

where y represents the unit output characteristic; x represents the unit input; ${\text{W}}_{\text{s}}$ represents a parameter to convert the input shape; $\left\{{\text{W}}_{\text{i}}\right\}$ represents the network transfer weight matrix.

#### 3.2.2 Soft thresholding

Soft thresholding is the core step of signal denoising. Through this formula (13), the features whose absolute value is less than a certain threshold can be deleted from the signal, and the features whose absolute value is greater than the threshold can be converged towards zero. The definition of threshold function is as follows:

$$y=\{\;\;\begin{array}{c} x-\tau \;\;\;\;\;\;\;\;\;\;x>\tau \\ 0\;\;\;\;\;\;\;-\tau \leq x\leq \tau \\ x+\tau \;\;\;\;\;\;\;\;x<-\tau \end{array}$$ (13)

The derivative of soft thresholding output concerning input is:

$$\frac{\partial y}{\partial x}=\{\;\;\begin{array}{c} 1\;\;\;\;\;\;\;\;\;\;\;\;\;\;\;\;\;x>\tau \\ 0\;\;\;\;-\tau \leq x\leq \tau \\ 1\;\;\;\;\;\;\;\;\;\;\;\;\;x<-\tau \end{array}$$ (14)

According to Eq. 14, the derivative of soft thresholding is 1 or 0. Therefore, the risk of gradient explosion of a network learning algorithm can be reduced.

#### 3.2.3 Attention mechanism

**FIGURE 4:**
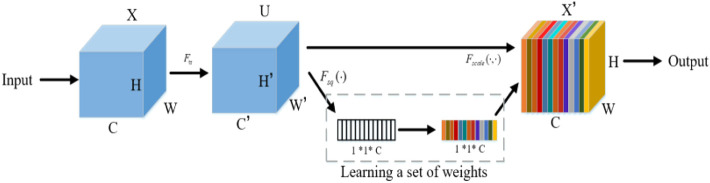
SENet network structure.

The attention mechanism can quickly scan global information, focus on useful information, and suppress useless information. In this paper, Squeeze and Congestion Network (SENet) is a novel deep learning algorithm for attention. The network is used to obtain the independent weight information of each group of samples, and the weight of the group is multiplied by the features of each channel, to adjust the feature size of each channel. In the SENet network, the process of obtaining weight includes global pooling, full connection layer, ReLU function, full connection layer, and Sigmoid function. The SENet network adopted in this paper scans global information and focuses on local key information. Its structure is shown in Figure 4. It is described by formula (15):

$$z_{c}=F_{sq}\left(u_{c}\right)=\frac{1}{W\times H}\overset{W}{\underset{i=1}{\sum }}\overset{H}{\underset{j=1}{\sum }}u_{c}\left(i,j\right)$$ (15)

#### 3.2.4 Soft thresholding based on SENet network

**FIGURE 5:**
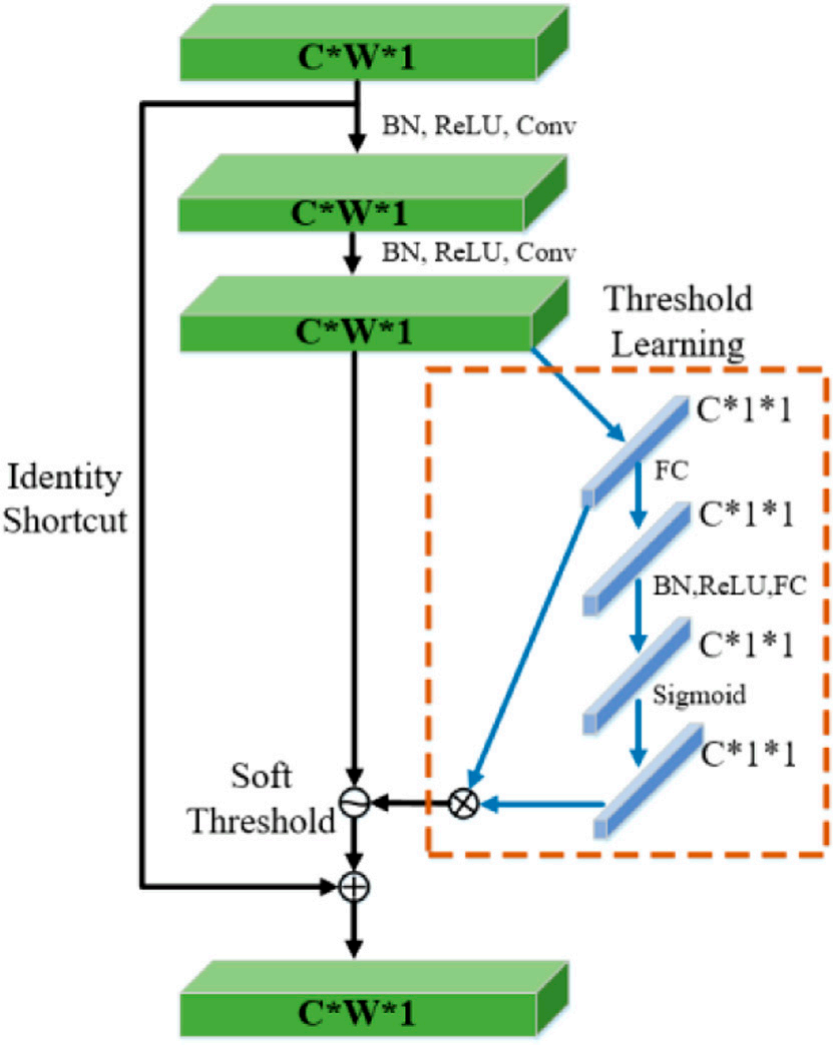
Detail of RSBU-CS unit structure.

The selection of feature threshold must meet three limitations: 1) The threshold is positive; 2) The threshold cannot be too large; 3) Set different thresholds for different samples. Considering the difference in irrelevant information contained in each group of input signals, the fixed threshold value cannot be selected, and the threshold value should be adaptively selected according to the noise situation. In this paper, the RSBU-CS unit is designed in DRSN to realize the learning of different channel thresholds by referring to the idea of the sub-network learning weight of SENet, as shown in Figure 5.

### 3.3 Muscle quality assessment

To date, there has been no clear evidence that the optimal number of neural network inputs. Selecting multiple input variables not only increases the training cost of the neural network but also has the risk of over-fitting. However, selecting too few input variables makes it difficult to build an accurate model and affects the prediction accuracy of modeling. Dombi et al. (1995) proposed the MIV index in 1995 to evaluate the impact degree of input neurons on output neurons in the neural network, and its absolute value represents the relative importance of influence. Therefore, we consider applying the MIV method to the DRSN regression model to improve the accuracy of interaction force prediction results. The calculation process is as follows:

(1) After the network training, add or subtract 10% of each independent variable characteristic ${\text{P}}_{\text{j}}$ in the training sample P based on its original value to form two new training samples P1 and P2 .
(2) Taking P1 and P2 as simulation samples and using the established network for simulation, two simulation results A1 and A2 were obtained.
(3) Calculate the difference between A1 and A2 , which is the Impact Value ( IV ) generated by changing the independent variable on the output.
(4) The independent variable can be obtained by averaging the IV value. For the MIV output by the dependent variable network, the independent variable whose cumulative value exceeds 90% can be found, that is, the selection of the input variable can be completed.

### 3.4 Model performance index

To verify the performance of our model compared with others, the MSE indicator is adopted to evaluate the performance of our model. MSE can be used to assess the degree of variation in the data and can demonstrate better accuracy of the prediction model. It can be described by formula (16):

$$MSE=\frac{1}{N}\overset{N}{\underset{i=1}{\sum }}{\left(x_{1,i}-x_{2,i}\right)}^{2}$$ (16)

where, $x_{1,\;i},\;x_{2,\;i}$ and N represents measure force, predicted force, and the total data.

## 4 Experimental results and analysis

**FIGURE 6:**
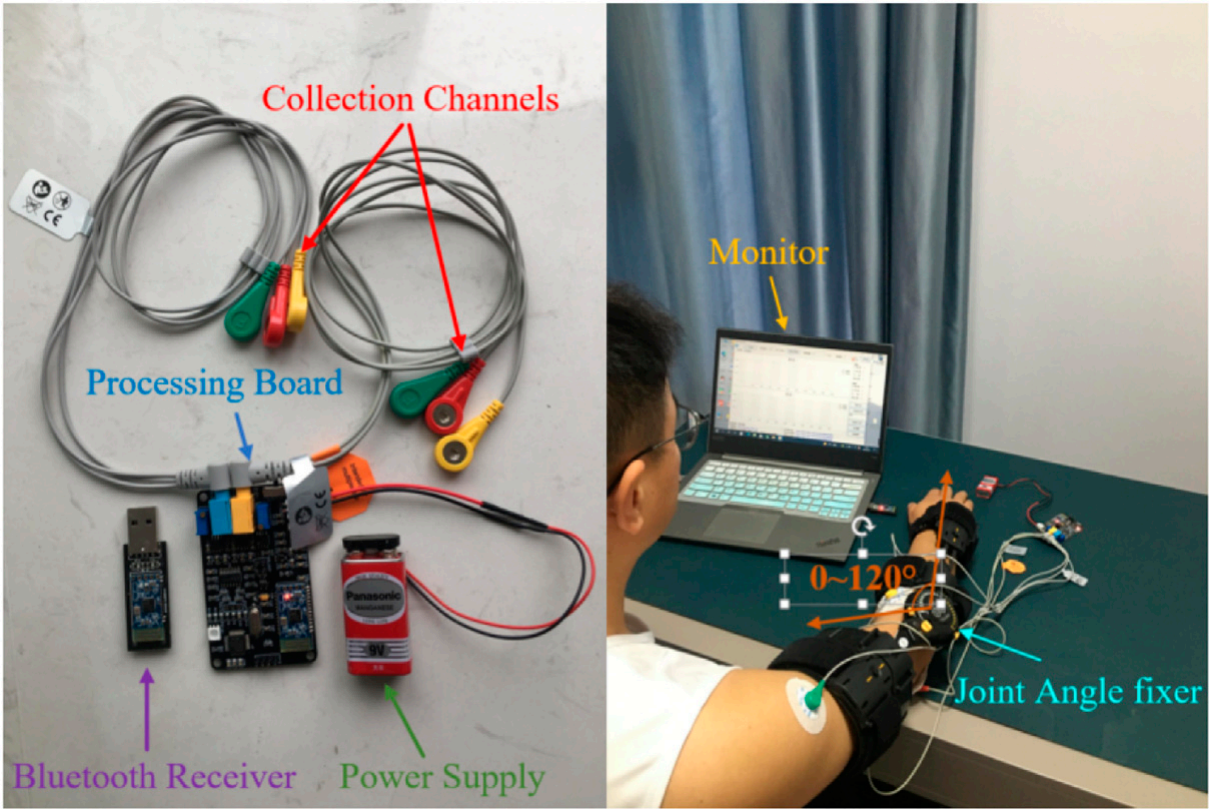
The experimental platform.

In the experimental part of this study, nine healthy adults were selected as subjects. All of them without mental or physical diseases. The detailed physical parameters are shown in Table 1. This experimental scheme was approved by the Institutional Review Committee of Hefei Institute of Physical Science, Chinese Academy of Sciences. All subjects knew the experiment procedure and signed informed consent before the experiment. 12 h before the experiment, there was no high-intensity exercise and the mood remained relaxed. They were asked to sit upright in a chair using Sichiray’s dual-lead electrical muscle sensor (Model: EDK0056, Sampling Frequency: 1,000 Hz, Voltage: 9V, Baud Rate: 115200bps). The experimental platform is shown in Figure 6.

**TABLE 1:** The physical parameters of each subject.

Subject	Gender	Age	Mass (kg)	Height (cm)
A1	Male	23	75	178
A2	Male	24	80	182
A3	Male	22	82	175
A4	Female	27	55	163
A5	Female	23	50	160

### 4.1 Dimension reduction results

**FIGURE 7:**
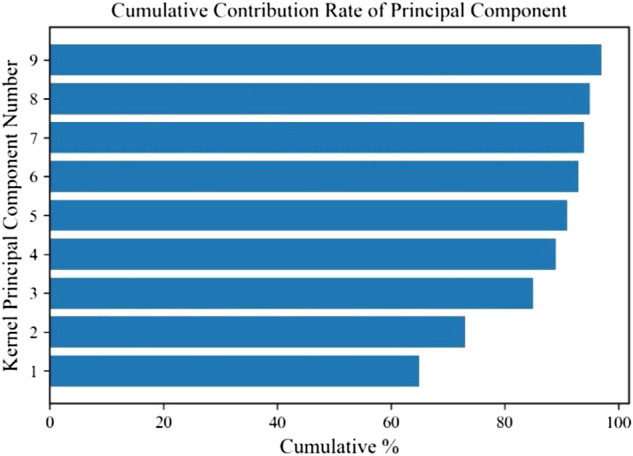
Cumulative contribution rate of the component.

In this experiment, we collected sEMG signals of muscles under elbow extension, elbow flexion, pronation, and pronation, and took biceps brachii as an example to reduce dimension through the KPCA algorithm. The cumulative contribution rate of the component is shown in Figure 7.

It can be seen that the cumulative contribution rate of the first three main components is more than 85%. The first principal component processed by KPCA can quickly reflect the changing trend of the sEMG signal. Therefore, compared with the original data, the dimension of the signal reconstructed by the first three principal components is significantly reduced.

### 4.2 Interaction force prediction comparison

**FIGURE 8:**
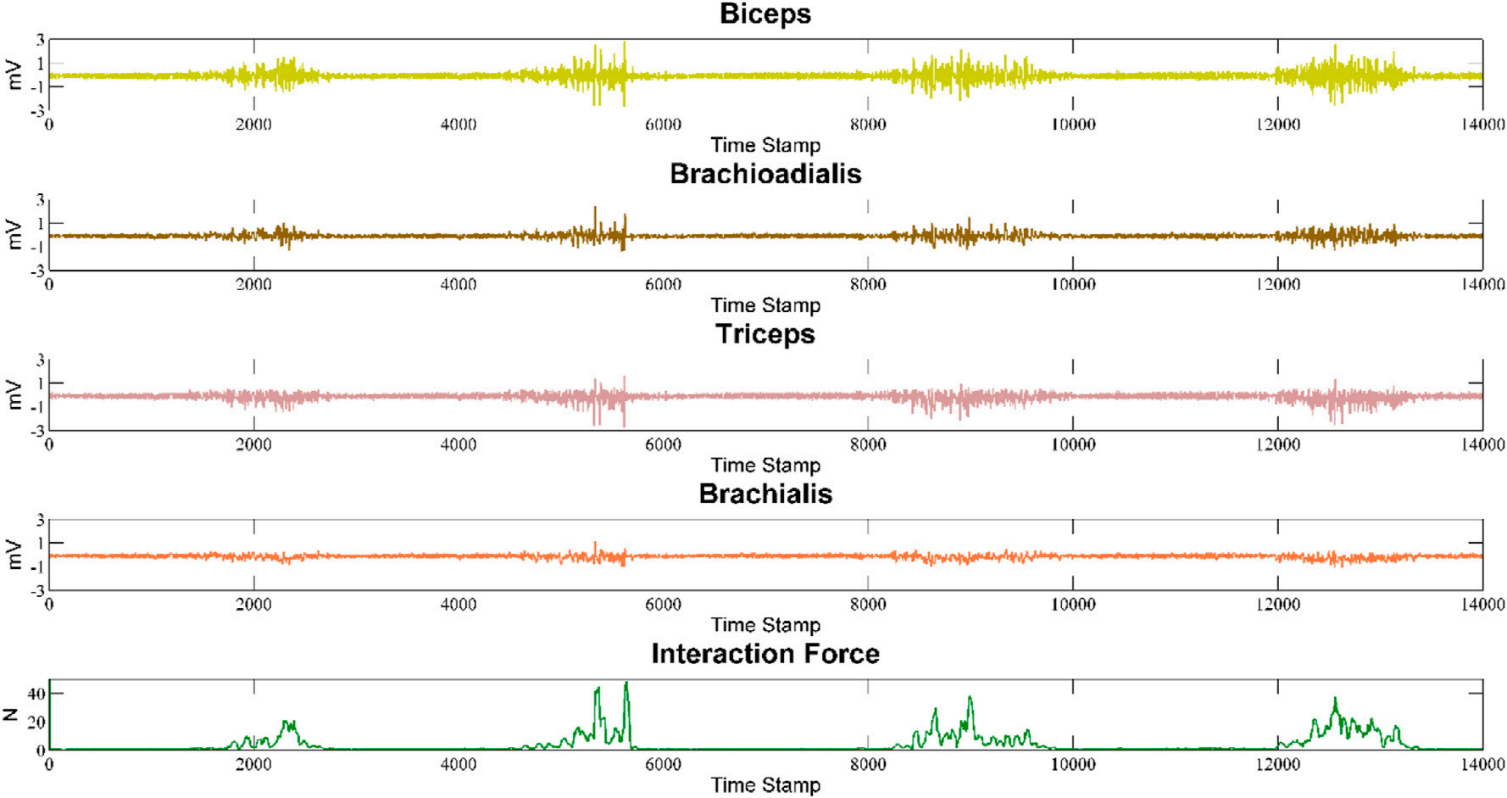
The raw EMG signal of the four channels and measured force.

In the force prediction experiment, the interaction force of the upper arm can be measured by the force sensor as the actual value. Each subject was required to perform the wrist force on the sensor at different angles for 2 s. The interaction force was determined by the feedback value of the sensor. The raw sEMG signal and force sensor feedback value of each channel are shown in Figure 8.

**FIGURE 9:**
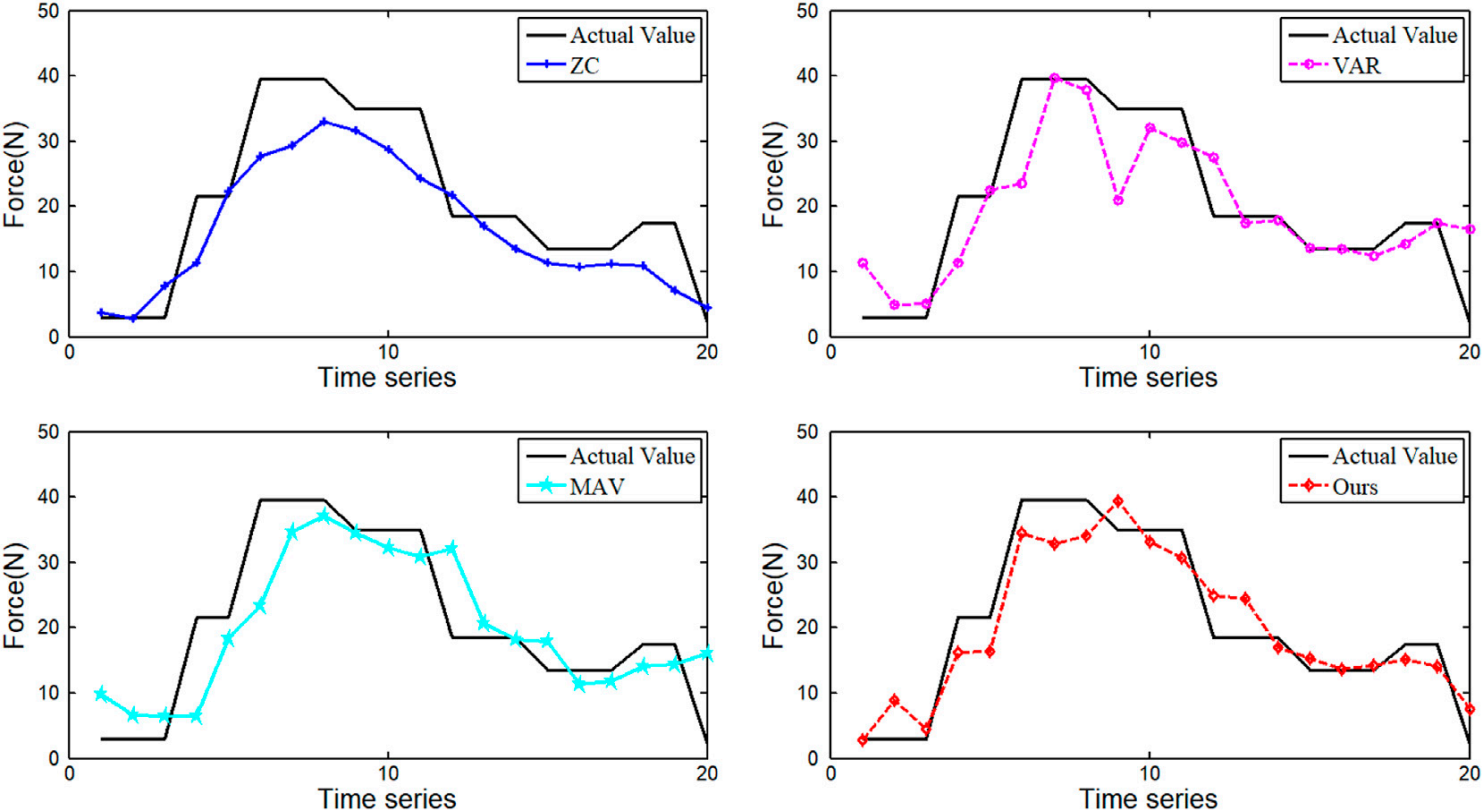
Comparison of interaction force prediction results under different features.

To verify the ability of the DRSN network to automatically extract signal features, three major time-domain features (MAV, ZC, and VAR) of EMG signals were extracted and fed into the network for prediction. The force prediction comparison results are shown in Figure 9.

As can be seen from Figure 9, the force prediction results based on MAV and VAR features are better than WA. The feature extracted from our method gets the best prediction result. In general, the forces estimated using these four features tend to be similar to the actual forces. However, the feature extracted from the DRSN model can achieve high prediction accuracy in the whole region.

In the meantime, we summarized the results of the other four subjects, as shown in Table 2. The results show that our method achieved the best performance in RMS , MAVE, and ρ indicators.

**TABLE 2:** Prediction results of different features for each subject.

Number		Interaction force		
		RMS(N)	MAVE(N)	ρ (%)
Subjects 1	MAV	0.87	0.57	99.32
	VAR	1.26	1.23	98.55
	ZC	2.69	1.64	96.23
	WA	1.30	1.03	98.35
	Ours	0.82	0.56	99.29
Subjects 2	MAV	0.76	0.59	99.67
	VAR	1.37	1.12	98.82
	ZC	2.81	1.39	96.90
	WA	1.38	1.01	98.03
	Ours	0.71	0.55	99.58
Subjects 3	MAV	0.70	0.65	99.53
	VAR	1.44	1.08	98.21
	ZC	2.76	1.54	97.12
	WA	1.41	0.98	98.65
	Ours	0.68	0.67	99.51
Subjects 4	MAV	0.74	0.62	99.34
	VAR	1.38	0.95	98.78
	ZC	2.72	1.59	96.12
	WA	1.47	0.94	98.76
	Ours	0.70	0.61	99.29
Subjects 5	MAV	0.70	0.57	99.45
	VAR	1.41	1.05	98.70
	ZC	2.68	1.55	96.51
	WA	1.31	0.90	98.68
	Ours	0.66	0.54	99.41

Furthermore, to verify the advantages of our method in accuracy and real-time performance, the MSE and prediction time index of the algorithm are compared, and the results are shown in Tables 3, 4.

**TABLE 3:** MSE comparison of different algorithms.

Movement	State-of-the-art algorithms			
	CNN	Informer	LSTM	Ours
Flexion	0.135	0.094	0.052	0.042
Extension	0.214	0.102	0.083	0.071
Pronation	0.199	0.195	0.054	0.069
Rotation	0.254	0.124	0.089	0.077

**TABLE 4:** Prediction time comparison of different algorithms.

Movement	State-of-the-art algorithms			
	CNN	Informer	LSTM	Ours
Flexion	0.0356	0.0145	0.0098	0.0058
Extension	0.0391	0.0215	0.0082	0.0049
Pronation	0.0489	0.0345	0.0096	0.0051
Rotation	0.0432	0.0298	0.0082	0.0055

We added different levels of noise to the signals respectively, and compared the MSE of the predicted results, as shown in Table 5. The results showed that the DRSN-based method had the best effect in suppressing noise redundancy. Compared with Table 3, the accuracy of prediction results after adding noise will decrease significantly, which further verifies the necessity of de-dimensionality reduction of data before prediction.

**TABLE 5:** MSE comparison with 5 db noise among different algorithms.

Movement	SNR (db)	State-of-the-art algorithms			
		CNN	Informer	LSTM	Ours
Flexion	5	0.598	0.359	0.248	0.095
Extension	5	0.631	0.487	0.378	0.121
Pronation	5	0.689	0.496	0.548	0.215
Rotation	5	0.732	0.498	0.568	0.326

**FIGURE 10:**
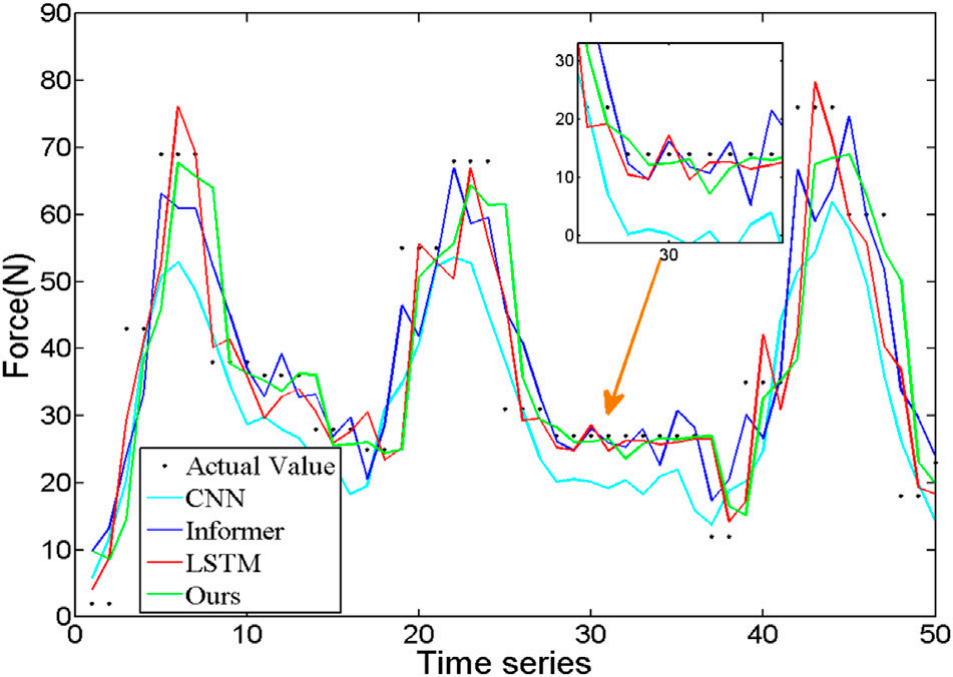
The comparison of interaction force prediction results of different algorithms.

**FIGURE 11:**
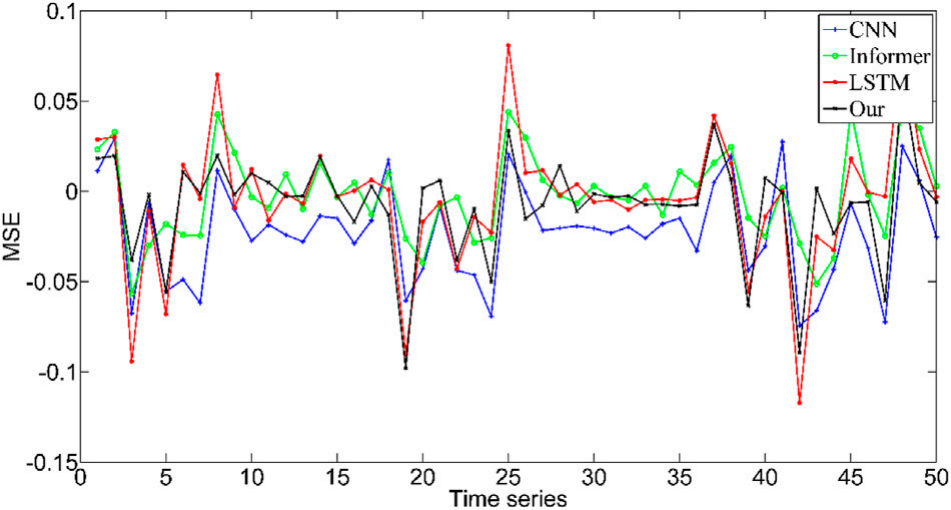
MSE index comparison results of different algorithms.

To compare the effectiveness of the proposed method in force prediction, we compare the current most advanced prediction algorithm, and the results are shown in Figure 10. Local zoom details are shown in the box in the Figure 10. The overall Mean Square Error (MSE) index comparison results as shown in Figure 11. Therefore, we can draw the following conclusions: 1.The accuracy of our method is superior to other state-of-the-art prediction algorithms during most time intervals. However, we find that the prediction performance of our model decreases in the time interval of [20 22] and [40 42]. The reason is that the motion state changes (from static to dynamic) in the interval. During this period, the collected EMG signal has redundant information due to the interference of motion artifacts, which would lead to the degradation of the tracking performance of the algorithm. How to eliminate the influence of motion artifacts on the prediction performance, which is the focus of our attention and the main task of our future work. 2. The proposed method in this paper got the best performance in terms of instantaneity compared with other models. Therefore, based on the above reasons, the proposed method still has greater advantages compared with other methods.

**FIGURE 12:**
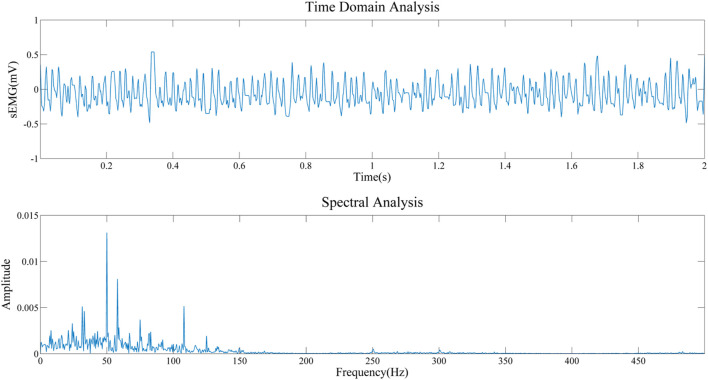
Frequency domain analysis in the relaxed state.

**FIGURE 13:**
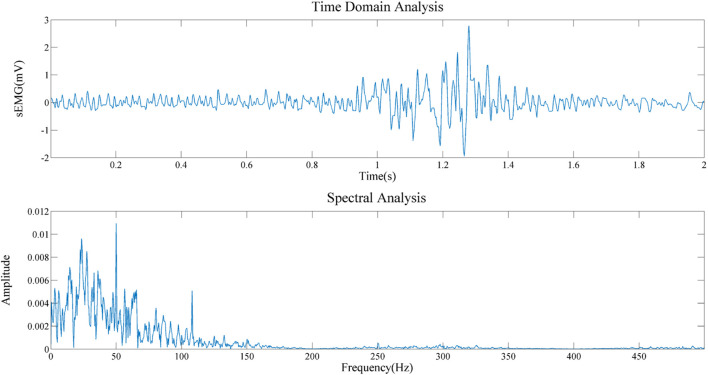
Frequency domain analysis in the static state.

To obtain the important information about the source signal in the frequency domain, including the frequency distribution, amplitude, and other information of the signal. Therefore, we conducted a spectral analysis of sEMG signals of biceps brachii muscle under static state and contraction states, as shown in Figures 12, 13. By comparing the two figures, we can draw the following conclusions: 1) The frequency distribution of EMG signal is 0–500 Hz, and its main frequency range is 0–150 Hz; 2) No matter in the relaxed state or in the contracted state, EMG signal will be obviously interfered by 50 Hz power frequency. 3) It can be seen that in the two states, it is obviously disturbed by signals with a frequency of about 110 Hz. We infer that the biceps brachii muscle is close to the cardiac, and this artifact is caused by ECG signal. This questions deserves further study in the future.

### 4.3 Analysis of muscles’ contribution to interaction force

**FIGURE 14:**
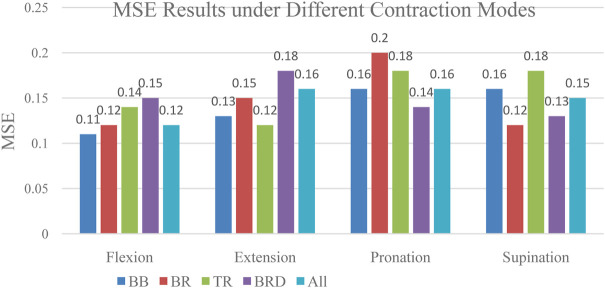
Prediction Results of Individual Muscle and Combined muscle Under Different Tasks.

To compare whether individual muscle or combined muscles is more suitable for interaction force prediction, we compared the interaction force prediction results of four individual muscles and combined muscles under four tasks. The MSE result was given in Figure 14. As can be seen from the figure, there are differences in the accuracy of the interaction force prediction results of EMG signals based on different muscles under different contraction modes. For example, in the situation of contraction, the prediction result based on BB muscle has the highest accuracy. Therefore, this muscle can be used as the main research object of force prediction under flexion contraction. The results provide meaningful guidance for the study of the contribution of other joint muscles.

**FIGURE 15:**
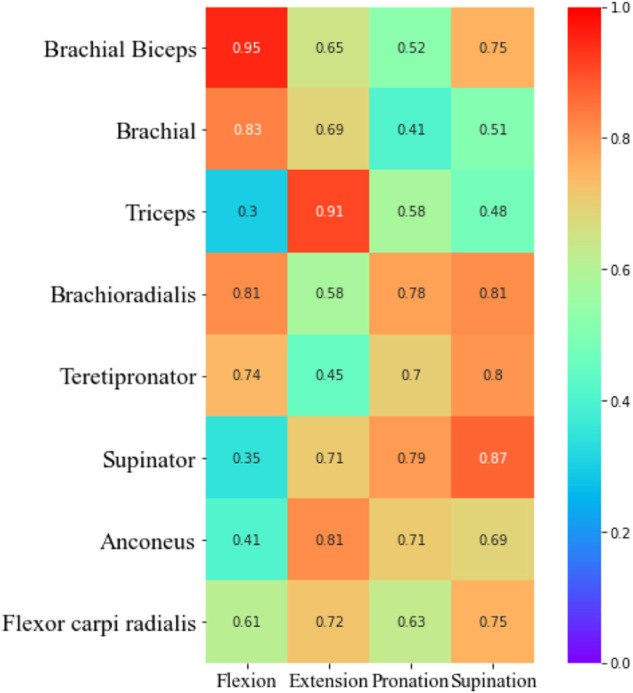
Heat map of individual muscles contribution.

As can be seen from the figure, the accuracy of prediction results based on each muscle, and combined muscles vary under different tasks. For example, in the flexion task, the MSE of prediction based on biceps muscle get the lowest. In the extension task, the MSE of the triceps muscle gets the lowest. In the pronation task, the MSE of the brachioradialis muscle gets the lowest. In the supination task, The MSE of brachialis gets the lowest. This finding revealed that it is not reliable to select only a certain muscle as a research object to predict the interaction force when performing different tasks. The muscle which contributes most to the interaction force should be chosen according to the task. Furthermore, we made a detailed analysis of the muscles of the upper limbs and summarized the contribution of each muscle based on the MIV index. The results are shown in Figure 15.

## 5 Conclusion and future works

In conclusion, this paper proposed an interaction force prediction framework and muscle collaborative quality assessment model based on the KPCA-DRSN model and MIV index. Firstly, by collecting the raw sEMG signals from multi-channel muscles of upper limbs, KPCA is adopted to achieve signal dimension reduction in consideration of its advantages in processing nonlinear signals. Effectively removing extraneous components from the signal. Then, the processed signals are directly input to the DRSN network for force prediction, which can effectively remove the redundant information irrelevant to tags and can automatically extract the effective features of the signal. Five healthy subjects were selected for several experiments, and the results show that compared with the state-of-the-art prediction algorithm, our method achieves the best performance in real-time and effectiveness. In addition, based on the MIV evaluation index, the muscle collaborative quality assessment model was constructed to explore the contribution of different muscles to the interaction force, which can provide effective guidance for muscle force prediction and intention recognition in the multi-muscle contraction task.

In the future, we will focus on the study of a compliant control scheme based on force prediction results, and finally, achieve the goal of human-machine integration. Future work of our study may include the following aspects:

• The upper limb motion intention recognition was realized based on the change of interaction force prediction results.
• Explore force prediction based on the fusion of biological signals (e.g., MMG signals, ECG signals, and EEG signals).
• The influence of ECG artifact on sEMG signals should be considered.

## Data Availability

The raw data supporting the conclusion of this article will be made available by the authors, without undue reservation.
